# Five Hundred Years of Mercury Exposure and Adaptation

**DOI:** 10.1155/2012/472858

**Published:** 2012-07-15

**Authors:** Guido Lombardi, Antonio Lanzirotti, Clifford Qualls, Francisco Socola, Abdul-Mehdi Ali, Otto Appenzeller

**Affiliations:** ^1^Laboratorio de Paleopatología, Cátedra Pedro Weiss, Universidad Peruana Cayetano Heredia, Lima, Peru; ^2^CARS, The University of Chicago, Bulding 434A, 9700 South Cass Avenue, Argonne, IL 60439, USA; ^3^Departments of Mathematics and Statistics, The University of New Mexico, Albuquerque, NM 87131, USA; ^4^Department of Medicine, Jackson Memorial Hospital, University of Miami, Miami, FL 33124, USA; ^5^Department of Earth and Planetary Sciences, Analytical Chemistry Laboratory, University of New Mexico, Albuquerque, NM 87131, USA; ^6^Department of Neurology, New Mexico Health Enhancement and Marathon Clinics Research Foundation, Albuquerque, NM 87122, USA

## Abstract

Mercury is added to the biosphere by anthropogenic activities raising the question of whether changes in the human chromatin, induced by mercury, in a parental generation could allow adaptation of their descendants to mercury. We review the history of Andean mining since pre-Hispanic times in Huancavelica, Peru. Despite the persistent degradation of the biosphere today, no overt signs of mercury toxicity could be discerned in present day inhabitants. However, mercury is especially toxic to the autonomic nervous system (ANS). We, therefore, tested ANS function and biologic rhythms, under the control of the ANS, in 5 Huancavelicans and examined the metal content in their hair. Mercury levels varied from none to 1.014 ppm, significantly less than accepted standards. This was confirmed by microfocused synchrotron X-ray fluorescence analysis. Biologic rhythms were abnormal and hair growth rate per year, also under ANS control, was reduced (*P* < 0.001). Thus, evidence of mercury's toxicity in ANS function was found without other signs of intoxication. Our findings are consistent with the hypothesis of partial transgenerational inheritance of tolerance to mercury in Huancavelica, Peru. This would generally benefit survival in the Anthropocene, the man-made world, we now live in.

## 1. Introduction

 The largest mercury processing in the Western Hemisphere was in Huancavelica in the Peruvian Andes, now a town of 30,000 people situated at 3676 m altitude. The extensive mercury deposits have been mined since pre-Hispanic times, they are just above the present town at ~4000 m. Although the mine closed 30 years ago, mining continues on small scales. The legacy of 500 years of mercury mining is now found in the city trenches and waterways [1], which are lined with mercury but this legacy is less evident in today's inhabitants of Huancavelica.

## 2. Historical Aspects

 Mining is a dangerous, unhealthy occupation but nowhere was it more health threatening than in Colonial South America, especially in Huancavelica.

 The widespread ecological impacts of mining in the Andes such as landscape destruction and pollution through the introduction of mercury and other toxicants into the biosphere were overshadowed by the population shifts to the then sparsely inhabited highlands. There, the challenges of survival in the ambient hypoxia of altitude, at which mining generally occurs in the Andes, were added to the deleterious health effects of mining.

 In 1928, Carlos Monge of Lima, Peru, described chronic mountain sickness (CMS) in a native miner from Cerro de Pasco, a mining town in Peru situated at 4338 m above sea level [2]. This syndrome is attributed to ambient hypoxia. It occurs in high altitude natives (hypoxia adapted individuals) who for, as yet, unknown reasons lose their adaptation to their hypoxic homeland. Recently, CMS was found to be associated with deranged expression of hypoxia-related genes [3]. In colonial times, less so now, mining required heavy physical labor which predisposes to CMS. The toxicity of mercury, when added to the stress of inescapable hypoxia at high altitude and the dangers to health and predisposition to injury of mining in colonial Huancavelica, earned the mine the infamous reputation as the “mina de la muerte” (the mine of death) [4].

 Because of its importance to the Spanish economy, mercury killed thousands of Andeans. Prior to the conquest, the locals used the Huancavelica cinnabar (mercury sulfide, HgS) deposits for body paint and cosmetics for their women. But, after the Spanish arrival, the lord of the local Angaraes people betrayed the cinnabar deposits to Amador de Cabrera in 1563. The riches of the deposits enticed newly arriving colonists to stake claims begin mining cinnabar, and distill mercury which they then sold to Mexico's silver refineries. Demand for mercury increased when the silver mines at Potosi began using mercury to amalgamate the ores in 1570 [4].

 Francisco de Toledo, a Spanish viceroy, arrived in Peru in 1569. He expropriated the Huancavelica mine. According to the new law the subsoil now belonged to the crown which granted its use to miners upon payment of taxes.

Under the new rules the Huancavelica treasury contracted with former claim holders to keep the mine operating, setting the amount of mercury to be produced and the price to be paid. The mercury was then resold to silver miners, at a profit [4].

 A change in the production of silver which required mercury for amalgamation of the ore increased the demand for labor in the mines. Toledo committed the colonial government to provide adequate manpower for the mercury producers. To do this, he copied Inca rules which required adult males to take turns on public projects called a “mit'a” (turn) and ordered neighboring provinces to send 900 “mitayos.” This number was increased to 3280 in 1577 [5]. The population in the surrounding areas decreased because of disease and the demands of the mita. This forced the government to reduce the size of the mita to 620 by 1645 and expand the area from which mitayos were committed into the labor force [4]. Mitayos received a low wage but served only for 2 months at a time, taken here as evidence for governmental awareness of the devastating health effects of mercury mining.

 Huancavelica became notorious as a dangerous workplace by the late 16th century and the Spanish crown received reports that the mita was abusive, unchristian, and unhealthy [6]. The high altitude of the mine added the stresses of ambient hypoxia, extreme temperatures and harsh weather to the hazards of mining, such as landslides and tunnel collapse. By 1600 the reputation of the Huancavelica mine as the “mine of death” was well deserved. The Franciscan Miguel Agia, in favor of the continuation of the mita, admitted that work in the mine was equivalent “to sending [Indians] to die.” And the “protector” of Indians Damián de Jeria likened the tunnel of the mine to a “public slaughterhouse” [7]. Luis de Velasco, another viceroy, wrote to the king about the health aspects of the Huancavelica mine in 1600:

*These quicksilver ores, when they extract them in the mines, they give out a dust that enters itself into the Indians as they breath and settles in the chest, of such evil quality, that it causes them a dry cough and light fever and at the end death without repair, because the doctors have it for an incurable evil.*

He wanted to close the mine but because of its economic importance, he pleaded for guidance from the crown [8, 9]. De Jeria wrote to the viceroy that although the mitayos had committed no crimes they were forced to endure conditions worse than those imposed on criminals in prisons and galleys. He argued that Huancavelica mining will eventually cause economic collapse because its continuance, costing so many lives of so many Indians… that which may be lost from not producing mercury is gained in the conservation of the Indians, for without them there will be neither quicksilver, nor silver, nor the common good, nor Peru [7].

 By 1630, the Count of Chinchón, then viceroy, wrote to the king about the mita:

*It is a most terrible matter because a free, innocent, defenseless, poor and afflicted people are condemned to a notorious risk of death their fear of the mita was such that trustworthy people who have seen it have reported to me that they take these miserable Indians by force against all their will from their houses and take them in iron collars and chains more than 100 leagues to put them in this risk… and from this has resulted their own mothers maiming and crippling their sons to preserve them [10].*

Pick-men and ore carriers developed silicosis (deposition of dust and silica particles in the lungs) rapidly because of enormous dust levels in the mine, but this also affected work in other mines. In Huancavelica, additionally, mercury killed by its inhalation. The miners remained in the mine throughout the week with little opportunity to wash. After returning to their huts, they transferred the dust to their living quarters and contaminated their families by prolonging exposure and mercury absorption through the skin and food.

 The symptoms of mercury poisoning were known to the workers who called it the “Huancavelica sickness” or the “evil of Huancavelica.” Although the usual treatment for poisoned miners was bloodletting to put “their bodily humors back into balance,” this increased their susceptibility to intercurrent illnesses. Eventually the graves of mitayos were said to contain puddles of mercury after their bodies decayed [4, 7]. The threat of mercury poisoning was even greater to those who worked the ovens to heat the ore and extract the mercury as the vapors cooled. And Indians were made to open the ovens before they had completely cooled to increase mercury extraction by reloading them with fresh ore. This practice enhanced their exposure to the toxic mercury vapors [4].

 Those rendered unable to work because of mercury poisoning were known as *azogados* (azogue = quicksilver). The symptoms were easily recognized. Weight loss, tremors, excessive salivation, ulcers of the mouth, restlessness, anemia, and eventually severe depression which often lead to alcoholism. Many miners had to be fed by their relatives because of the violent tremors which could not be controlled to bring food to their mouths.

 The Prince of Santo Buono (viceroy 1716–1720) urged the abolition of the mita. Although initially rejected by the crown and the Council of the Indies it was finally approved on humanitarian grounds. By 1740 the mining guild began using blasting powder to dislodge the ore. Skilled miners were now able to stand and swing sledge hammers in the mining shafts which, by now, had acquired improved ventilation.

 Ulloa, governor of Huancavelica, wrote in 1760 [11] that miners were now free of mercury intoxication because the ore contained so little quicksilver but poisoning still occurred at the ovens when workers failed to let them cool completely before reloading them.

 The miners had useful remedies for the symptoms of mercury poisoning. Ulloa reported that azogados descended to warmer lowlands, drank huge amounts of *chicha*, a beer made from maize, worked very hard in the fields, and “sweated” out the mercury. The documented improvement was, likely, due to the diuretic effect of the beer which expelled the mercury concentrated in the kidneys. After some weeks of heavy drinking and hard physical labor the shakes, excessive salivation and mouth ulcers disappeared and some miners returned to work in the Huancavelica mine.

 Although the mine was especially deadly because of its mercury, most Spaniards at that time thought that the Indians were destined to die anyway if this was necessary to obtain the metal. Camargo wrote to the king in 1595 [12]:

*They are barbarous people and without knowledge of God; in their lands they are only occupied in idolatries and drunkenness and other vices of great filthiness… These people going to work the mines first seems to me a service to God and to your Holy Catholic Royal Majesty and for the good of the natural Indians themselves, because the mines they teach them doctrine and make them hear mass and they deal with Spaniards by which they become ladinos [Hispanicized]…. losing their barbarity, and they have no place for the idolatries of their lands by being outside of those sites where they do them and being occupied, which is the principal thing.*

Camargo affirmed his “great desire to [also] die in the service of Your Holy Catholic Royal Majesty” [4].

 Some improvements in health occurred with new mining methods but these failed to take into account the huge pollution of the biosphere of Huancavelica which persists to the present. The refining ovens were along the river where the escaping vapors settled on the ground, in the water, and blew across town depositing mercury on everything. These vapors were converted into methyl mercury by bacterial activity which then easily entered the food chain and which is especially injurious to the nervous system.

## 3. Epidemics of Mercury Poisoning

 Outbreaks of mercury poisoning have occurred sporadically in more recent times. They were often associated with industrial pollution. These outbreaks were traced to methyl mercury, the highly toxic form of mercury [13]. The best-described mass poisoning occurred in Minamata Bay, Japan, in the 1950s [14]. But intoxications restricted to single families due to ingestion of mercury with longterm consequences have also been reported [15].

 Mercury poisoning in infants (pink disease, also known as acrodynia) associated with the use of teething powders containing mercury, especially in England and Australia, has been eliminated by the removal of the metal from these remedies. Clinical features of acrodynia, were autonomic “hyperactivity” characterized by acral vasodilatation, excessive sweating and tachycardia. Pain, paresthesias, and hypotonia allowed the assumption of characteristic postures by the affected infants [16]. These symptoms reflect the especially injurious effects of mercury on the autonomic nervous system (ANS). The link between mercury and acrodynia is complex. The syndrome has not been found in recent epidemics of mercury poisoning in Japan, but it reemerged in an epidemic of methyl mercury (used as a fungicide) poisoning employed for the sterilization of diapers in Argentinean babies (for review see [16]).

## 4. Mercury in Human Hair

 Mercury compounds occur in the environment and their levels are rising due to anthropogenic activities, especially the burning of fossil fuels. Coal contains large amounts of mercury, which is released into the atmosphere upon burning and it is then transported, possibly, around the globe. Even low concentrations of mercury can cause subclinical effects [17], and slightly increased levels of mercury in hair are associated with decreased academic performance [18]. The World Health Organization (WHO) places the level at which toxicity occurs at 50 ppm of mercury in hair and advises that healthy people should have no more than 5 ppm of mercury in their hair. However, normal mercury levels in hair are difficult to come by because they are dependent on the amount and type of fish eaten, on location (especially where the environmental mercury burden may be high) on the sampling methods, and on analytical methods [19]. Thus, in populations that have been exposed to high burdens of mercury for centuries, like those in Huancavelica, the effects of such exposure are difficult to assess.

## 5. Hair: Biologic Aspects

 Hair is renewed throughout life; it has a well-defined variability in growth influenced by diet, age, blood flow to the skin, and hormonal and metabolic signals which are, ultimately, regulated by the ANS. But hair also reflects biologic rhythms because of clock-like signals that affect its growth through the intermediary of the ANS [20].

 Recurrent circa-annual periods of slow and fast rhythms in the hydrogen isotope ratios of human and animal hair can be recognized. Using power spectral analysis of the ratios along the length of the hairs can reveal the effects of life at altitude and toxin accumulation on biologic rhythms [21].

 Here we report a study of hydrogen isotope ratios in the hair and of heart rate variability in the same residents of Huancavelica, Peru, to assess their biologic rhythms and ANS function. We carried out this study to determine whether centuries of ancestral and present exposure to increased mercury in the biosphere could affect ANS function and give evidence for continuing deleterious effects on the health in modern Huancavelica residents.

## 6. Results

 The demographics of the 5 subjects and mercury and copper levels in their hair are given in Table 1. Because the length of the hair reflects the duration of the stored information on biologic rhythms, our study was confined to women who generally have longer hair. The mercury levels varied from none detectable to 1.041 ppm. We confirmed these very low levels in the hair from two Huancavelica subjects using the X26A hard X-ray microprobe at the Brookhaven synchrotron. No detectable mercury in single hairs from each subject was reported by the X-ray microprobe.

To strengthen our conclusions about the surprisingly low mercury levels in the hair in Huancavelica residents we used a control from the USA.

In Table 1 cold vapor atomic absorption spectrometry was used to measure total mercury (organic + inorganic) and inductively coupled plasma-atomic emission spectrometry for the other element (one sample was misplaced by the analytical company performing the assays no values for Hg are available; here labeled lost).

**Figure 1:**
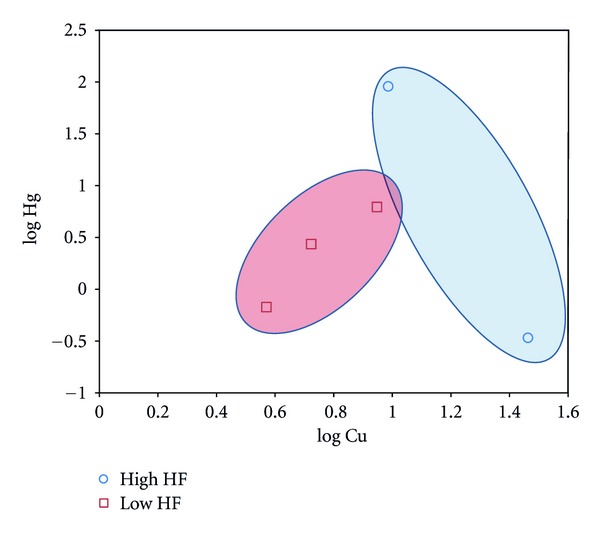
The high-frequency/low-frequency power bands of the hydrogen isotope ratios along the hair reflect the control by the autonomic nervous system (ANS) of biologic rhythms. Hg and Cu both influence the power bands. Note that Cu is associated with low-HF power of the spectra of the hydrogen isotope ratios along the length of the hairs.

 To assess the impact of individual metals on the hydrogen isotope ratios of low frequency/high frequency (LF/HF) power bands, a measure of ANS control of biologic rhythms, we constructed “impact graphs” shown in Figure 1. These power bands were affected by both copper and mercury levels in the hair clearly separating the LF and HF power according to mercury and copper levels.

**Figure 2:**
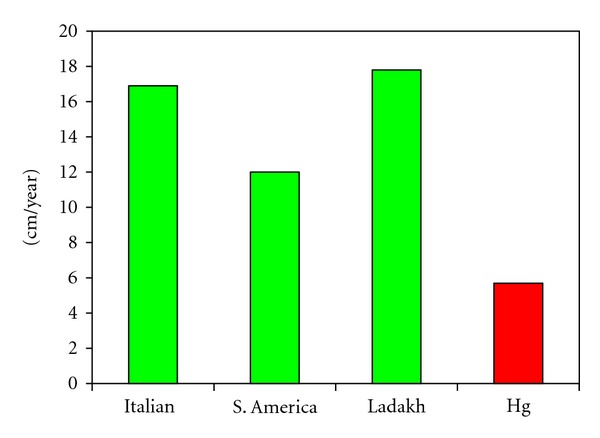
Growth rates of human scalp hair from different locales and from Huancavelica (Hg). Significantly slower yearly growth (~6 cm/year) is evident in modern residents of Huancavelica.

The annual growth rate reported for normal human hair is ~16 cm/year [21]. In our controls (*n* = 4) as determined from the sinusoidal variation in hydrogen isotope ratios in our samples from the USA and Europe, it was 14.2 ± 3.7 SD cm/year. The growth rate of hair from Huancavelica (*n* = 5) was 6.4 ± 1.7 SD cm/year (*P* < 0.001) (Figure 2).

**Figure 3:**
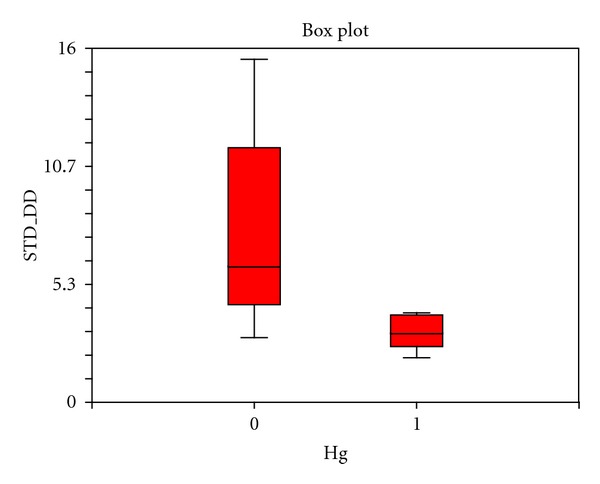
Box plots showing the variance (standard deviation; STD) of hydrogen isotope ratios along the length of the hair in Huancavelica residents (1) and controls (0). The statistically significant reduced variance in Hg exposed individuals (*P* = 0.01) signifies deranged biologic rhythms with significant reduction of variance in the system's control by the autonomic nervous system.

The standard deviation of the variance of the power spectra of the hydrogen isotope ratios between controls from other parts of the world and that from Huancavelica is shown in Figure 3. Remarkably, this variance is significantly less in Huancavelica residents (*P* = 0.01).

**Figure 4:**
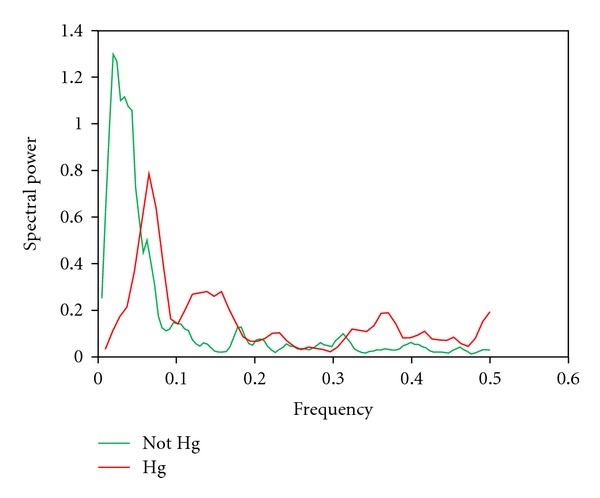
Pooled power spectra derived from hydrogen isotope ratios. There is statistically significant less low power (*P* < 0.05) in the mercury (Hg) spectrum (red) derived from Huancavelica hair.

 Pooling of hydrogen isotope power spectra from Huancavelica and control hair is shown in Figure 4. The only statistically significant difference between these spectra is in the low-power frequencies (*P* < 0.05), though the power of high frequencies appears also to be less (NS).

**Figure 5:**
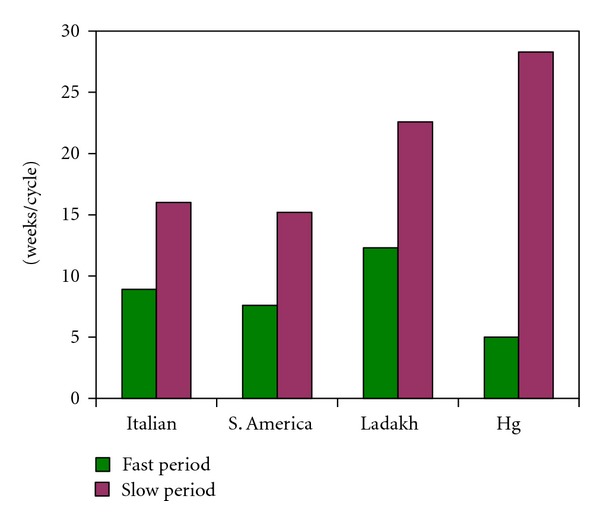
Fast and slow periods of hair growth in weekly cycles. The hairs from Huancavelica (Hg) show a shorter fast period and very prolonged slow period which correspond to the rest phase of hair growth.

 The annual growth rates of hair can be shown to occur in fast and slow periods of weekly cycles (Figure 5). The shorter fast period, and very prolonged slow period which correspond to the rest phase of hair growth, are consistent with the reduced annual hair growth in Huancavelica residents.

**Figure 6:**
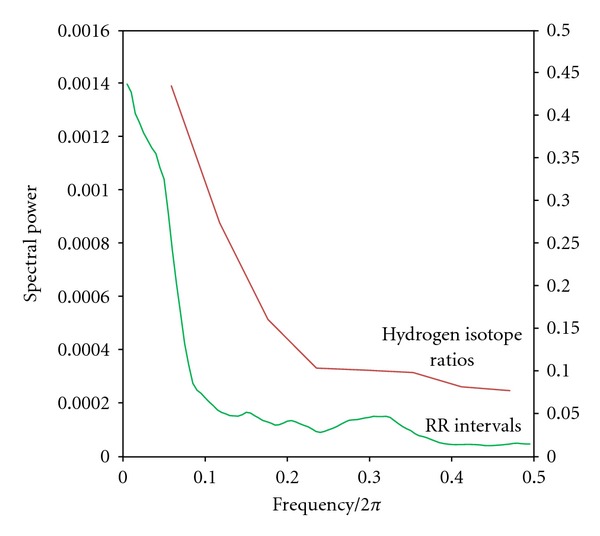
Comparison of power spectra derived from hydrogen isotope ratios along the length of the hairs and power spectra derived from heart rate intervals (RR) in the same subjects. Note that the low- and high-frequency power bands are of comparable lengths but the spectral power appears different (NS) (“stacked” averaged spectra from all subjects). Red line hydrogen isotope ratios; green line heart rate variability (RR intervals). Spectral power, on the *y*-axis, is hydrogen isotope ratios (right) and RR intervals (left).

 We compared the power spectra derived from heart rate variability with those obtained from the hydrogen isotope ratios in the hair, in the same subjects (Figure 6). The low- and high-power frequency bands are of comparable length but, not surprisingly, the spectral power is different because of the different time scales—heart rate variability in milliseconds, hydrogen isotope ratios in days.

**Figure 7:**
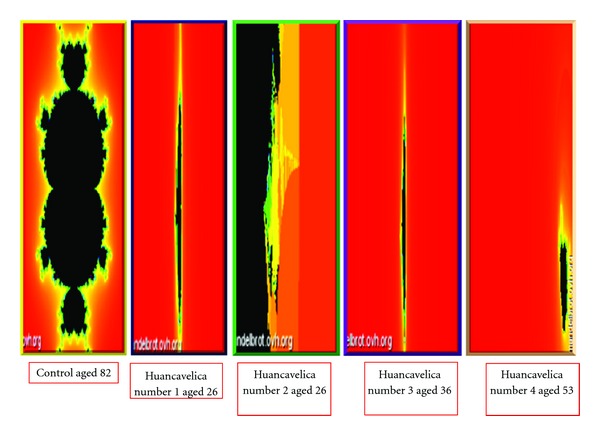
Mandelbrot sets showing the complex interactions between Hg and biologic rhythms. Notable is the lack of structural features in the sets derived from all Huancavelica residents in keeping with the lack of variability found in the power spectra derived from the hydrogen isotope ratios of the same individuals (see Figure 3).

Because of the complexity of the interactions between mercury and biologic rhythms as derived from hydrogen isotope ratios along the length of the hairs we used fractal analysis to illustrate the differences between those living in a mercury-polluted environment and a control (Figure 7).

These results are consistent with severe dysfunction of the ANS evidenced also by the reduced annual hair growth in Huancavelica residents.

## 7. Discussion

The most famous mercury poisoned character was the “Mad Hatter,” the one from the Mad Tea Party in *Alice in Wonderland*. He was partner to the March Hare, mad as only a March hare can be (though the hare was not poisoned by mercury). Lewis Carroll created the characters. However, the phrases “mad as a hatter” and “mad as a March hare” were in common use in 1837, well before the publication of Alice in Wonderland in 1865.

Hatters did go mad because felt used for making hats was cured by mercurious nitrate and the mercury vapors inhaled by hatters gave them the characteristic symptoms reported also by azogados (quicksilver poisoned miners from Huancavelica) centuries earlier who noted the shakes, hallucinations, and psychosis as found in hatters centuries later.

 Mercury was used in Europe throughout medieval times for medicinal purposes or as mercury sulphide, an ingredient of the bright red ink used by scribes in monasteries. Increased levels were found in medieval bones of those who had either employed it for the treatment of leprosy or syphilis, or with increased occupational exposure such as pharmacists [22].

Mercury is a neurotoxin but its damaging effects on humans are unpredictable and dependent, in part, on its chemical speciation and interactions with other metals in the environment and in the diet. Organic mercury which is the primary source of mercury exposure is principally derived from the diet.

 Huancavelica is listed amongst the ten most polluted places in the world [1] and despite its listing, our measurement of mercury levels in the hair of current Huancavelica residents was noticeably low (Table 1) and, clinically, Huancavelica residents showed no signs of mercury poisoning as recorded in hatters and azogados centuries earlier. However, measurements of mercury levels in the hair are not good indicators of mercury's toxicity. While levels of mercury can be accurately measured in the blood, the hair has no blood supply and the levels derived from hair represent metabolism over time and reflect the chronobiology of the individuals rather than the blood levels of this metal. In modern times, hair is also frequently washed with shampoos and dried with hair driers; these manipulations can add or remove mercury and thus compound the difficulties of assaying the mercury levels in the hair [23].

 Here we show that people living in a heavily mercury-polluted environment, in Huancavelica, may have acceptable mercury levels, by international standards, in their hair and no overt clinical symptoms of intoxication. Nevertheless, the effects of mercury exposure are evident from the reduced rate of hair growth and disturbed biologic rhythms in these people. Taken together these findings give unequivocal evidence of the toxicity of mercury to the human autonomic nervous system the most vulnerable part of the nervous system to mercury's damage, in modern Huancavelica, Peru.

 The high-frequency/low-frequency (HF/LF) ratios are measures of the control by the autonomic nervous system (ANS) of biologic rhythms [21]. We find that Hg and Cu both affected the power bands of the HF and LF. Mercury does interact with other metals to produce its effect on biologic rhythms in Huancavelica and such interactions might account for the unpredictable effects of Hg on individual physiology in other parts of the world too (Figure 1).

 The growth rate of human scalp hair is variable but on average ~16 cm/year. The growth rate of hair in Huancavelica is significantly reduced compared to our controls. We suggest that the altered biologic rhythms in modern Huancavelica residents may have contributed to this failure to renew the hair at an appropriate rate (Figure 2).

 The standard deviation of the variance of the power spectra of the hydrogen isotope ratios along the length of the hairs between our controls from other parts of the world and that from Huancavelica is shown in Figure 3. This variance is significantly less in Huancavelica residents (*P* = 0.01). Normal rhythmic oscillations in biology are marked by high variability (large variances); less variability degrades performance of the system [24]. The reduction in variance we observed is yet another expression of the deranged biologic rhythms in our Huancavelica subjects.

 The low-frequency peak of the power spectra reflects primarily the sympathetic nervous system's control of autonomic variability. A decrease or absence of this peak in heart rate variability and in the power spectra derived from hydrogen isotope ratios in the hair in the same individuals is seen in ANS diseases such as pure autonomic failure (PAF), manifested by widespread failure of sympathetic nervous system function [21]. Our identification of significant reduction in low-frequency power in the hydrogen isotope ratios in Huancavelica subjects (Figure 4) further supports ANS impairment in these individuals.

 Long-term rhythms recurring at weekly, monthly, or yearly intervals are well known in animal and human physiology. The recurring annual fast periods reflect growth while the slow periods reflect the rest stages in hair growth. The shortened fast period rhythms in Huancavelica residents' hair (Figure 5) are in keeping with the reduced hair growth rate and further support the altered ANS function (and biologic rhythms) in these people.

 To confirm our findings from hydrogen isotope derived biologic rhythms, we measured heart rate variability using 2 minutes of recording of cardiac action (~150 heart beats) in the same subjects (Figure 6). Averaged power spectra showed comparable durations of low- and high-frequency bands and spectral power did not differ significantly. We have previously shown that spectra obtained from human heart rate variability were comparable to those derived from power spectra obtained from hydrogen isotope ratios in the hair and from growth lines from teeth [25]. Our results confirmed that biologic rhythms as gleaned from power spectral analysis reflected the pulsing of life, the varying rhtymicity of physiologic functions even when the ANS has been affected by disease or mercury as in Huancavelica residents.

 Fractals are evident throughout nature. They reflect recurrent relations at each point in a space. They can show complex natural phenomena in graphic form such as ice crystals on a window pane and the intricate contours of mountains and ridges, and river branching. In biology they illustrate the 3/4 scaling of metabolism with body size, the complexity of heart rate control, of blood circulation, growth, development, and lifespan [26]. The term fractal was introduced by Benoit Mandelbrot in 1982. Here we used Mandelbrot fractal sets to show the complex relationships between Hg, its effects on the ANS's control of human biologic rhythms, and the variability of hydrogen isotope ratios along the length of the hair in our subjects (Figure 7). The fractals derived from all Huancavelica subjects clearly reflect derangement in biologic rhtymicity and the lack of variance which characterizes their exposure to Hg, supporting our contention of continuing influence of this metal on human life in modern Huancavelica.

 Taken together our results show that survival in a heavily, mercury, polluted environment is possible without overt signs of mercury intoxications such as those found during mercury epidemics of the past or in miners during the Spanish exploitation of Peru's mineral riches. Nevertheless, careful analyses of the function of the most mercury vulnerable part of the nervous system gives evidence of continued effects in contemporaneous Huancavelica residents.

 How can we reconcile the clear evidence of Hg's effects on biologic rhythms and ANS function with the unexpected low levels of the metal in the hair of these same individuals and their apparent lack of overt signs of intoxication? First, there is the fact that Hg in hair is a poor indicator of its possible toxic effects. Secondly, we propose a parallel between how human height and life expectancy increase in a short span of ~300 years after the industrial revolution [27]. This well-documented phenomenon has been explained by several contributing factors such as technological advances and physiological adaptation [28].

The history of mining in the high Andes is replete with anecdotes of how technical advances in the extraction of ores improved miners' survival [11] and at the same time, in Huancavelica, increased the yield of Hg extraction. While the cause of the shortened lifespan in Huancavelica miners remained a mystery for long, nevertheless, the miners themselves devised useful adaptations which allowed them to return to work after total incapacitation, extending their lifespan [11]. Just like in the early years of the industrial revolution in Europe, improved health, better nutrition through obtaining the means of continuing to work, increased productivity of the mines and of the miners themselves, implied greater resilience, increase in longevity, and improved fertility in the high Andes. Human genetic potentials that could produce drastic changes in the relatively short span of 12–15 generations in Europeans during the industrial revolution may also have been operative in the mining towns of the high Andes for more than 500 years. The Andean miners had the opportunity to adapt to enormous environmental pollution and now their descendants tolerate, relatively, but not entirely, unscathed, the persistent Hg in the Huancavelica biosphere. Nevertheless, our multilevel analyses clearly showed that even 21 generations is insufficient for complete adaptation to the horrendous mercury pollution.

 How could such adaptation have occurred? We propose that an experimental model may have recently been described in fruit flies [29]. The microbiome, the symbiotic bacteria in the gut of the flies, profoundly affected their physiology. The molecular mechanisms that underlie the interactions of gut bacteria with their host are beginning to be unraveled in the flies [29].

In humans the microbiome is also closely linked to physiology and disease. Mercury resistant bacteria have been well recognized [30]. Since one route of entry of mercury in humans is through the gut, the microbiome might be the first to encounter the toxin and the bacteria adapt to increasing levels of ingested mercury. The survival of a functioning microbiome in the human gut like in the flies [29] is, in turn, closely linked to the survival of the host. Adaptation over a period of some 500 years to enormous environmental mercury loads in Huancavelica may have ultimately been the results of bacterial (microbiome) adaptation to mercury.

 For this proposal it is necessary to invoke so-called transgenerational effects. These have been found in humans and animals [31]. In Sweden, for example, the nutritional and smoking habits of paternal grandparents could influence the health of their grandchildren and in Australia the mercury sensitivity of paternal grandparents who had pink disease as infants, affected their grandchildren [32]. This hypothesis is eminently testable in contemporaneous Huancavelica residents.

 A third possible explanation is found in the theory of tolerance to disease or to environmental stressors such as toxins [33]. This theory has the most support from infectious diseases, where tolerance to bacterial invaders has been shown to offer evolutionary advantages by avoiding the costly means to eliminate the infectious agents. In this setting, tolerance to invaders offers a defense strategy that favors survival in heavily contaminated surroundings. Similarly, cellular responses to stress can offer survival advantages in stressful environments such as the hypoxia of altitude or excessive heat by inducing reactive oxygen species (ROS) or activating numerous heat-shock proteins, respectively.

 Resistance and tolerance are thought to be complimentary strategies for living [33] also in heavily polluted environments such as in Huancavelica. Analyzing the molecular pathways which allow relatively normal survival in a heavily mercury, polluted environment after five hundred years of mercury exposure may offer insights into strategies of coping with the climate and environmental changes wrought by the anthropocene, the man-made world, we now live in.

## 8. Materials and Methods

 The ethics committee of the New Mexico Health Enhancement and Marathon Clinics (NMHEMC) Research Foundation reviewed and approved the study. Informed written consent was obtained from all participants.

The hair was obtained from five Huancavelica females.

### 8.1. Study Limitations

This was a field study, and as such suffers from limitations common to such studies such as small number of subjects, poor adherence to study protocols, and low number of controls. Nevertheless, wherever statistical analyses were possible these were performed and gave clear and meaningful results. (See Text S1 in Supplementary Material available online at doi:10.1155/2012/472858).

### 8.2. Microfocused Synchrotron X-Ray Fluorescence Analyses

 Microfocused synchrotron X-ray fluorescence analyses were performed at beamline X26A at the National Synchrotron Light Source, Brookhaven National Laboratory, Upton, NY, USA. Beamline X26A utilizes a bending magnet source on the NSLS 2.8 GeV electron storage ring, which operates at a current of 300 mA. For these experiments a monochromatic X-ray beam was used, monochromatized using a Si(111) channel-cut monochromator and tuned to an incident beam energy of 12.5 keV. The beam was focused to a spot size of 5 *μ*m in the vertical *x* 8 *μ*m in the horizontal using a pair of dynamically bent, grazing-incidence mirrors, each 100 mm long, arranged in a Kirkpatrick-Baez (KB) geometry. X-ray fluorescence from the human hairs was measured using a combined set of three energy dispersive detectors; a Canberra 9-element HPGe array detector placed 90° to the incident beam within the plane of the storage ring to minimize backgrounds from Compton scattering. The other two detectors are single element Radiant Vortex-EX silicon drift diode detectors also at 90° to the incident beam, but each sitting 45° above and below the plane of the storage ring, respectively. The 9-element HPGe array provides an active area of 900 mm^2^ and each silicon drift diode detector has an active area of 50 mm^2^. All eleven detector elements are integrated simultaneously using the XMap series of compact PCI-based digital spectrometers produced by X-ray Instrumentation Associates (XIA) interfaced through EPICS and controlled through in-house client software written in IDL. Incident beam intensity was monitored using an ion-chamber upstream of the focusing optics and all images were corrected for changes in incident beam flux through normalization to the change in ion-chamber counts over time.

### 8.3. Hydrogen Isotope Ratios

Hydrogen isotope ratios were determined using the continuous-flow high-temperature-reduction technique [34]. Hair was wrapped in silver foil and placed into the combustion chamber of a mass spectrometer using a Carlo Erba AS 200-LS autosampler. Three mm long sections of hair were sampled beginning at the proximal end. The stable isotopic compositions of low-mass elements such as hydrogen are reported as “delta” (Ð) values in parts per thousand (‰). Ð  values are calculated as follows: in (‰) = (*R* sample/*R* standard − 1) 1000, where “*R*” is the ratio of the heavy to the light isotope in the sample. The stable isotope standard for hydrogen is reported relative to the Standard Mean Ocean Water (SMOW). Isotope composition is reported in relation to this standard which has been defined as 0‰  [33].

### 8.4. Heart Rate Intervals

These were obtained from 2 minute EKG recordings using standard leads. Approximately 150 RR intervals were measured for each subject. The statistical analysis was similar to that used for the hydrogen isotope power spectra analysis (see below) and the frequency bands were identical to those chosen for the hydrogen isotope bands of the power spectra derived from the hair of the same subject.

### 8.5. Mandelbrot Sets

Data derived from each hair were entered into the Mandelbrot set-online generator by Dawid Makiela (http://www.Mandelbrot.ovh.org/).

*X*_1_: mercury content; *Y*_1_: low-frequency/high-frequency ratio of power spectrum; *X*_2_: elemental content (Cu); *Y*_2_: total power of low, mid- and high frequencies; *X*:   total low power; *Y*: total high power; *R*: sum of low, mid, and high frequencies of the power spectrum obtained from the hydrogen isotope ratios along the length of each hair.

The functions were *Z*_*n*+1_ = *Z*_*n*_^2^ + *Z*_0_. Maximum number of iterations was 100.

To strengthen our conclusions about biologic rhythms derived from the hair in Huancavelica residents we used an aged control from the USA. The variance of biologic rhythms decreases with advancing age [35] thus an old control subject, US resident, should serve better as a standard for comparison to the biologic rhythms derived from the hair of young mercury exposed subjects from Huancavelica.

### 8.6. Statistical Methods

In order to determine the relationship between metals in hair and the hydrogen isotope spectra in hair, we computed Pearson and Spearman correlations of each metal with measures of the spectra. The log-transformed quantities of Hg and Cu were found to be related to the spectral power in the high-frequency band We identify two clusters of high-frequency power (low and high power) in the Hg-Cu scatter plot of the 5 Huancavelica subjects (see Figure 1).

#### 8.6.1. Growth Rate of Hair Calculations

There is a clear annual sinusoid in hydrogen isotope ratios in hair. The fact that periodicity of this sinusoid is 52 weeks allows us to compute annual growth rates in centimeters. Fitting the annual sinusoid for Huancavelica subject #3 by nonlinear regression yielded the function of length along the hair in cm:

(1) Predicted  dD=−91.1315+1.5832∗sin⁡ (.8850∗cm+1.3243).

The frequency of the sinusoid is 0.885 radians/cm. One can use the frequency of the fitted sinusoid to estimate the growth rate that matches the 0.0193 cycles/week = 1 cycle/52 weeks, whose periodicity is 52 weeks. So (freq/2*π*)*(growth/year)/(52 weeks/year) set equal to 1 cycle/52 weeks implies the following:

(2) growthyear=2π(frequency  of  fitted  annual  sinusoid).

Here growth/year = 2*π*/.885 = 7.1 cm/year. The average growth rate for the 5 Huancavelica subjects was 6.4 ± 1.7 SD cm/year. This is compared to the average growth rate of ~16 cm/year in human hair by a one-sample *t*-test (see Figure 2).

#### 8.6.2. Spectral Variance

All power spectra were computed using the finite Fourier transform, decomposes time series into a sum of sine and cosine waves of different amplitudes and wavelengths (PROC SPECTRA from SAS). The spectral variance for a given time series of hydrogen isotope ratios in hair is the total power (area under the periodogram). The comparison of these standard deviations (square root of the variance) for the 5 Huancavelica subjects to our sample of controls are made by a two-sample *t*-test (see Figure 3).

The spectral power in the low-frequency band and their standard errors are computed as described in Priestly [36]. These are then compared by *t*-test (see Figure 4).

#### 8.6.3. Periodicities

Fast and slow periods of hair growth in weekly cycles is computed from the frequencies of higher power observed in the periodogram. The form of the calculation is as follows:

(3) Period=dt(freq)(growth),

where dt is the increment of the series (see Figure 5).

For Figure 6, the power spectra for both heart rate interval (RR) and hydrogen isotope ratio time series measured in the same 5 Huancavelica subjects (the 5 series are stacked) are superimposed.

## Supplementary Material

Microfocused synchrotron X-ray fluorescence analyses.

## Figures and Tables

**Table 1:** Analyses of metals in hair from control and subjects in Huancavelica, Peru.

Sample ID	Cu (mg/K)	Hg (*μ*g/L)	Hg (mg/Kg) (ppm)
Control age 82	3.72	0.673	*0.002*
Number 1 age 26	29.0	*ND*	*ND*
Number 2 age 26	8.86	6.23	*0.129*
Number 3 age 36	9.68	90.9	*1.041*
Number 4 age 48	7.48	lost	*lost*
Number 5 age 53	5.29	2.73	*0.058*
