# Supplementary material for: Five Hundred Years of Mercury Exposure and Adaptation
Source: J Biomed Biotechnol. 2012 Jul 15;2012:472858. doi: 10.1155/2012/472858 (PMC3403783; doi:10.1155/2012/472858)
Supplement: Supplementary file 1 — Microfocused synchrotron X-ray fluorescence analyses. [file 472858.f1.pdf]

Supplemental on line material text S1

Microfocused synchrotron X-ray fluorescence analyses

Microfocused synchrotron X-ray fluorescence analyses were performed at beamline X26A at the National Synchrotron Light Source, Brookhaven National Laboratory, Upton, NY, USA. Beamline X26A utilizes a bending magnet source on the NSLS 2.8 GeV electron storage ring, which operates at a current of 300 mA. For these experiments a monochromatic X-ray beam was used, monochromatized using a Si(111) channel-cut monochromator and tuned to an incident beam energy of 12.5 keV. The beam was focused to a spot size of 5 μm in the vertical x 8 μm in the horizontal using a pair of dynamically-bent, grazing-incidence mirrors, each 100 mm long, arranged in a Kirkpatrick-Baez (KB) geometry. X-ray fluorescence from the mammoth hairs was measured using a combined set of three energy dispersive detectors; a Canberra 9-element HPGe array detector placed 90° to the incident beam within the plane of the storage ring to minimize backgrounds from Compton scattering. The other two detectors are single element Radiant Vortex-EX silicon drift diode detectors also at 90° to the incident beam, but each sitting 45° above and below the plane of the storage ring respectively. The 9-element HPGe array provides an active area of 900 mm^2 and each silicon drift diode detector has an active area of 50 mm^2. All eleven detector elements are integrated simultaneously using the XMap series of compact PCI-based digital spectrometers produced by X-ray Instrumentation Associates (XIA) interfaced through EPICS and controlled through in-house client software written in IDL. Incident beam intensity was monitored using an ion-chamber upstream of the focusing optics and all images were corrected for changes in incident beam flux through normalization to the change in ion-chamber counts over time.

Hydrogen isotope ratios

Hydrogen isotope ratios were determined using the continuous-flow-high-temperature-reduction Technique [1]. Hair was wrapped in silver foil and placed into the combustion chamber of a mass spectrometer using a Carlo Erba AS 200-LS auto-sampler. Three mm long sections of hair were sampled beginning at the proximal end. The stable isotopic compositions of low mass elements such as hydrogen are reported as "delta" (Ð) values in parts per thousand (‰). Ð values are calculated: in (‰)=(R sample/R standard-1)1000 where "R" is the ratio of the heavy to the light isotope in the sample The stable isotope standard for hydrogen is reported relative to the **S**tandard **M**ean **O**cean **W**ater (SMOW). Isotope composition is reported in relation to this standard which has been defined as 0‰. [1]

Heart rate intervals

We used the same methodology for heart rate intervals (RR intervals) as that for the analysis of the power spectra derived from hydrogen isotope ratios along the length of the hairs. In these calculations hydrogen intervals were substituted by RR intervals (see below).

Mandelbrot sets

The Mandelbrot set consists of a number of points; it does not represent anything more than these points. The images generated are not just in the Mandelbrot set but are also outside the points of interest and are colored in such a way as to represent how long it took to decide that a point was not in the set.

A Mandelbrot set is generated by a mathematical operation such that for each point c in some region of a complex plane the X value represents the "real" part of a complex number and the Y

value represents the "imaginary" part of the complex number. The operation consists by repeating the following equation: $z_{n+1} = z_n^2 + c$ (where $z_0 = 0$). If the absolute value of z_n remains within the bounds, then the point c is in the Mandelbrot set. If, z_n goes to infinity, then the point remains outside the bounds of the set.

The colors of the set are dependent on the number of calculations, here 100 iterations. The Mandelbrot set is not a simple shape, but is irregular. The set is named after Benoit Mandelbrot, who coined the term "fractal" for these irregular shapes.

Data derived from each hair were entered into the Mandelbrot set-online generator by Dawid Makiela© (Mandelbrot.ovh.org)

X_1: Mercury content; Y_1: Low frequency/high frequency ratio of power spectrum; X_2: elemental content (Cu); Y_2: total power of low, mid- and high frequencies; X: total low power; Y: total high power; R: sum of low, mid and high frequencies of the power spectrum obtained from the hydrogen isotope ratios along the length of each hair.

The Functions were $Z_{n+1}=Z_n^2+Z_0$. Maximum number of iterations was 100.

To strengthen our conclusions about biologic rhythms derived from the hair in Huancavelica residents we used an aged control from the USA. The variance of biologic rhythms decreases with advancing age [2] thus an old control subject, US resident, should serve better as a standard for comparison to the biologic rhythms derived from the hair of young mercury exposed subjects.

Statistical Methods

In order to determine the relationship between metals in hair and the hydrogen isotope spectra in hair, we computed Pearson and Spearman correlations of each metal with measures of the spectra. The log-transformed quantities of Hg and Cu were found to be related to the spectral power in the high frequency band We identify two clusters of high frequency power (low and high power) in the Hg-Cu scatter plot of the 5 Huancavelica subjects (see Fig 1).

Growth Rate of Hair Calculations.

There is often a clear annual sinusoid in hydrogen isotope ratios in hair. The fact that periodicity of this sinusoid is 52 weeks allows us to compute annual growth rates in centimeters. Fitting the annual sinusoid for Huancavelica subject #3 by nonlinear regression yielded the function of length along the hair in cm:

Predicted dD = **-91.1315** +**1.5832***sin(**.8850***cm+**1.3243**)

The frequency of the sinusoid is 0.885 radians/cm. One can use the frequency of the fitted sinusoid to estimate the growth rate that matches the 0.0193 cycles/week = 1 cycle/52 weeks, whose periodicity is 52 weeks. So (freq/ 2π)*(growth/year)/(52 weeks/year set equal to 1 cycle/ 52 weeks implies

growth/year = 2π /(frequency of fitted annual sinusoid).

Here growth/year = 2π / .885 = 7.1 cm/year. The average growth rate for the 5 Huancavelica subjects was 6.4±1.7 SD cms./year. This is compared to the average growth rate of 16 cms/year in human hair by an one-sample t-test (see Figure 2).

Spectral Variance.

All power spectra were computed using the finite Fourier transform, decomposes time series into a sum of sine and cosine waves of different amplitudes and wavelengths. (PROC SPECTRA from SAS). The spectral variance for a given time series of hydrogen isotope ratios in hair is the total power (area under the periodogram). The comparison of these standard deviations (square root of the variance) for the 5 Huancavelica subjects to our sample of controls are made by a two-sample t-test (see Figure 3).

The spectral power in the low frequency band and their standard errors are computed as described in Priestly [3] (Spectral Analysis and Time Series, Academic Press, 1981, 7th printing 1992, page 427). These are then compared by t-test (see Figure 4).

Periodicities.

Fast and slow periods of hair growth in weekly cycles is computed from the frequencies of higher power observed in the periodogram. The form of the calculation is

$$Period = \frac{dt}{(freq)(growth)}, \text{ where } dt \text{ is the increment of the series}$$

(see Figure 5).

For Figure 6, the power spectra for both heart rate interval (RR) and hydrogen isotope ratio time series measured in the same 5 Huancavelica subjects (the 5 series are stacked) are superimposed.

References

1. Anonymous. (2006) USGS Menlo Park California Resources on Isotopes. Fundamentals of Stable Isotope Geochemistry.
2. Appenzeller O, Oribe E. (1997) The Autonomic Nervous System; An introduction to basic and clinical concepts. 5$^{th.}$ Edition Elsevier, Amsterdam, pp.910, 517-518.
3. Priestly M. Spectral Analysis and Time Series, Academic Press, 1981, 7th printing 1992, page 427.
